# Supplementary material for: Glycemic Index, Glycemic Load and Dyslipidemia in Adolescents from Chiapas, Mexico
Source: Nutrients. 2024 May 14;16(10):1483. doi: 10.3390/nu16101483 (PMC11124452; doi:10.3390/nu16101483)
Supplement: Supplementary file 1 [file nutrients-16-01483-s001.zip › nutrients-2969615-supplementary.pdf]

Table S1. Top food contributors to dietary GI and its association with abnormal blood lipids (Odds Ratios and 95% Confidence intervals).

Variables		Food items									
		Tortillas (maize)	Traditional baked goods	Sweetened Soft Drinks	Rice	Sugar	Cookies	White Bread	Lemonade or fruit water with sugar	Pasta	Juices, industrialized
	% of dietary GI	**38.7**	**6.6**	**5.0**	**4.4**	**3.9**	**3.7**	**3.3**	**3.1**	**2.6**	**2.4**
Total cholesterol ≥ 200 mg/dL	Model 1[a]	0.97 (0.94 - 1.01)	0.98 (0.94 - 1.01)	0.99 (0.97 - 1.02)	1.01 (0.99 - 1.04)	1.00 (0.97 - 1.02)	1.02 (0.99 - 1.05)	1.01 (0.99 - 1.03)	1.00 (0.97 - 1.04)	0.99 (0.95 - 1.03)	0.99 (0.98 - 1.02)
	Model 2[b]	0.97 (0.94 - 1.01)	0.98 (0.94 - 1.02)	0.99 (0.97 - 1.02)	1.01 (0.99 - 1.04)	1.00 (0.97 - 1.02)	1.02 (0.99 - 1.05)	1.01 (0.99 - 1.03)	1.00 (0.96 - 1.04)	0.98 (0.93 - 1.03)	0.99 (0.98 - 1.02)
	Model 3[c]	0.96 (0.93 - 1.00)	0.98 (0.95 - 1.02)	0.99 (0.97 - 1.02)	1.02 (0.99 - 1.04)	1.00 (0.98 - 1.02)	1.03 (1.00 - 1.06)	1.01 (0.99 - 1.03)	1.00 (0.96 - 1.04)	0.98 (0.93 - 1.03)	0.99 (0.98 - 1.02)
HDL-c <40 mg/dL	Model 1[a]	1.00 (0.98 - 1.02)	1.01 (0.99 - 1.02)	1.00 (1.00 - 1.01)	1.00 (0.99 - 1.01)	1.01 (1.00 - 1.02)	0.99 (0.98 - 1.00)	1.00 (0.99 - 1.01)	1.00 (0.98 - 1.01)	1.01 (1.00 - 1.02)	1.00 (0.99 - 1.02)
	Model 2[d]	1.00 (0.98 - 1.02)	1.01 (0.99 - 1.02)	1.00 (0.99 - 1.01)	1.00 (0.98 - 1.01)	1.01 (1.00 - 1.02)	0.99 (0.98 - 1.01)	1.00 (0.99 - 1.01)	1.00 (0.98 - 1.01)	1.01 (1.00 - 1.02)	1.00 (0.99 - 1.02)
	Model 3[e]	1.00 (0.98 - 1.02)	1.01 (0.99 - 1.02)	1.00 (0.99 - 1.01)	1.00 (0.98 - 1.01)	1.00 (0.99 - 1.01)	0.99 (0.98 - 1.00)	1.00 (0.99 - 1.01)	1.00 (0.98 - 1.01)	1.01 (1.00 - 1.02)	1.00 (0.99 - 1.02)
LDL-c ≥ 110 mg/dL	Model 1[a]	0.98 (0.95 - 1.01)	0.97 (0.94 - 1.01)	1.00 (0.98 - 1.02)	1.01 (0.99 - 1.03)	0.99 (0.97 - 1.01)	1.01 (0.99 - 1.04)	1.02 (0.99 - 1.03)	1.03 (0.99 - 1.06)	0.98 (0.94 - 1.02)	1.01 (0.99 - 1.05)
	Model 2[b]	0.98 (0.95 - 1.01)	0.97 (0.94 - 1.01)	1.00 (0.98 - 1.02)	1.01 (0.99 - 1.03)	0.99 (0.97 - 1.01)	1.02 (0.99 - 1.04)	1.02 (0.99 - 1.04)	1.03 (0.99 - 1.06)	0.97 (0.93 - 1.02)	1.01 (0.99 - 1.05)
	Model 3[f]	0.97 (0.93 - 1.00)	0.98 (0.95 - 1.02)	1.00 (0.98 - 1.03)	1.01 (0.99 - 1.04)	0.99 (0.97 - 1.01)	1.02 (1.00 - 1.05)	1.01 (0.99 - 1.04)	1.03 (0.99 - 1.06)	0.98 (0.94 - 1.02)	1.01 (0.99 - 1.05)
Triglycerides ≥ 130 mg/dL	Model 1[a]	1.00 (0.97 - 1.02)	0.99 (0.97 - 1.00)	1.00 (0.99 - 1.01)	0.99 (0.97 - 1.00)	1.00 (0.99 - 1.01)	1.01 (1.00 - 1.02)	1.00 (0.99 - 1.01)	1.00 (0.98 - 1.01)	1.01 (0.99 - 1.02)	0.99 (0.98 - 1.01)
	Model 2[b]	1.00 (0.97 - 1.02)	0.99 (0.97 - 1.00)	1.00 (0.99 - 1.01)	0.99 (0.97 - 1.00)	1.00 (0.99 - 1.01)	1.01 (1.00 - 1.02)	1.00 (0.99 - 1.01)	1.00 (0.98 - 1.01)	1.01 (0.99 - 1.02)	0.99 (0.98 - 1.01)
	Model 3[g]	0.99 (0.97 - 1.01)	0.99 (0.98 - 1.00)	1.00 (0.99 - 1.01)	0.99 (0.97 - 1.00)	1.00 (0.99 - 1.01)	1.02 (1.00 - 1.03)	1.00 (0.99 - 1.01)	1.00 (0.98 - 1.02)	1.01 (0.99 - 1.02)	0.99 (0.97 - 1.01)

[a] Adjusted for sex. [b] Adjusted for sex and body fat percentage (normal vs. high). [c] Adjusted for sex, body fat percentage (normal vs. high) and energy-adjusted protein intake (grams per day). [d] Adjusted for sex and language of the mother (Spanish vs. Indigenous). [e] Adjusted for sex, language of the mother (Spanish vs. Indigenous) and protein intake (percentage of total energy intake per day). [f] Adjusted for sex, body fat percentage (normal vs. high) and protein intake (percentage of total energy intake per day). [g] Adjusted for sex, body fat percentage (normal vs. high) and energy-adjusted dietary fiber intake (grams per day).

Table S2. Top food contributors to dietary GL and its association with abnormal blood lipids (Odds Ratios and 95% Confidence intervals).

Variables		Food items									
		**Tortillas (maize)**	**Traditional baked goods**	**Sweetened Soft Drinks**	**Rice**	**Sugar**	**Cookies**	**Lemonade or fruit water with sugar**	**White Bread**	**Juices, industrialized**	**Pasta**
	**% of dietary GL**	**39.6**	**6.8**	**5.1**	**4.3**	**3.8**	**3.6**	**3.0**	**2.9**	**2.7**	**2.7**
Total cholesterol ≥ 200 mg/dL	Model 1[a]	0.99 (0.97 - 1.01)	0.97 (0.94 - 1.01)	0.98 (0.93 - 1.04)	1.02 (0.99 - 1.05)	1.02 (0.92 - 1.12)	1.01 (0.98 - 1.05)	1.00 (0.97 - 1.04)	1.01 (0.97 - 1.04)	0.96 (0.94 - 1.03)	0.98 (0.91 - 1.06)
	Model 2[b]	0.99 (0.97 - 1.02)	0.98 (0.94 - 1.02)	0.99 (0.94 - 1.04)	1.02 (0.99 - 1.05)	1.03 (0.93 - 1.15)	1.02 (0.98 - 1.05)	1.01 (0.96 - 1.05)	1.00 (0.96 - 1.04)	0.96 (0.95 - 1.03)	0.97 (0.89 - 1.06)
	Model 3[c]	0.99 (0.97 - 1.02)	0.98 (0.94 - 1.02)	0.99 (0.94 - 1.04)	1.02 (0.99 - 1.05)	1.03 (0.93 - 1.15)	1.02 (0.98 - 1.05)	1.01 (0.96 - 1.05)	1.00 (0.96 - 1.04)	0.96 (0.95 - 1.03)	0.97 (0.89 - 1.06)
HDL-c <40 mg/dL	Model 1[a]	1.00 (0.99 - 1.01)	1.02 (1.00 - 1.03)	1.00 (0.99 - 1.02)	0.99 (0.98 - 1.01)	1.03 (0.98 - 1.08)	0.99 (0.97 - 1.01)	0.99 (0.97 - 1.01)	0.99 (0.97 - 1.01)	1.00 (0.98 - 1.03)	1.02 (0.99 - 1.05)
	Model 2[d]	1.00 (0.99 - 1.01)	1.02 (1.00 - 1.03)	1.00 (0.99 - 1.02)	0.99 (0.98 - 1.01)	1.03 (0.98 - 1.08)	0.99 (0.97 - 1.02)	0.99 (0.97 - 1.01)	0.99 (0.97 - 1.01)	1.00 (0.98 - 1.03)	1.02 (0.99 - 1.05)
	Model 3[e]	1.00 (1.00 - 1.01)	1.01 (1.00 - 1.03)	1.00 (0.98 - 1.02)	0.99 (0.98 - 1.01)	1.02 (0.98 - 1.07)	0.99 (0.97 - 1.01)	0.99 (0.97 - 1.01)	0.99 (0.97 - 1.01)	1.00 (0.98 - 1.03)	1.02 (0.99 - 1.05)
LDL-c ≥ 110 mg/dL	Model 1[a]	0.99 (0.97 - 1.01)	0.96 (0.90 - 1.01)	0.99 (0.95 - 1.03)	1.02 (0.99 - 1.05)	0.95 (0.82 - 1.11)	1.01 (0.97 - 1.04)	1.04 (0.99 - 1.08)	1.02 (0.99 - 1.06)	0.95 (0.94 - 1.02)	0.98 (0.90 - 1.05)
	Model 2[f]	0.99 (0.96 - 1.01)	0.96 (0.90 - 1.02)	0.99 (0.95 - 1.03)	1.02 (0.99 - 1.04)	0.96 (0.81 - 1.14)	1.02 (0.98 - 1.06)	1.04 (0.99 - 1.09)	1.02 (0.99 - 1.05)	0.93 (0.92 - 1.01)	0.96 (0.89 - 1.04)
	Model 3[g]	0.98 (0.95 - 1.01)	0.97 (0.91 - 1.03)	0.99 (0.95 - 1.04)	1.02 (0.99 - 1.05)	0.97 (0.81 - 1.16)	1.02 (0.99 - 1.06)	1.04 (0.99 - 1.10)	1.02 (0.99 - 1.05)	0.93 (0.91 - 1.01)	0.97 (0.89 - 1.05)
Triglycerides ≥ 130 mg/dL	Model 1[a]	1.00 (1.00 - 1.01)	0.98 (0.96 - 1.00)	0.99 (0.97 - 1.01)	0.98 (0.96 - 1.00)	0.98 (0.94 - 1.03)	1.02 (1.00 - 1.04)	0.99 (0.97 - 1.01)	1.00 (0.98 - 1.02)	0.97 (0.94 - 1.01)	1.02 (0.99 - 1.05)
	Model 2[f]	1.01 (1.00 - 1.01)	0.98 (0.96 - 1.00)	0.99 (0.97 - 1.01)	0.98 (0.96 - 1.00)	0.99 (0.94 - 1.04)	1.02 (1.00 - 1.04)	0.99 (0.97 - 1.01)	0.99 (0.97 - 1.02)	0.97 (0.93 - 1.01)	1.02 (0.99 - 1.05)
	Model 3[h]	1.00 (0.99 - 1.01)	0.98 (0.97 - 1.00)	0.99 (0.97 - 1.02)	0.98 (0.96 - 1.01)	0.99 (0.94 - 1.04)	1.03 (1.01 - 1.06)	1.00 (0.98 - 1.03)	1.00 (0.98 - 1.03)	0.97 (0.93 - 1.01)	1.02 (0.98 - 1.05)

[a] Adjusted for sex. [b] Adjusted for sex and weight status (underweight or normal vs. overweight or obesity). [c] Adjusted for sex, weight status (underweight or normal vs. overweight or obesity) and protein intake (percentage of total energy intake per day). [d] Adjusted for sex and mother´s education (<6 years vs. ≥6 years). [e] Adjusted for sex, mother´s education (<6 years vs. ≥6 years) and energy-adjusted dietary fiber intake (grams per day). [f] Adjusted for sex and body fat percentage (normal vs. high). [g] Adjusted for sex, body fat percentage (normal vs. high) and protein intake (percentage of total energy intake per day). [h] Adjusted for sex, body fat percentage (normal vs. high) and energy-adjusted dietary fiber intake (grams per day).
